# Fertility and Pregnancy Outcomes in Patients With Adenomyosis: Is Adenomyosis Synonymous With Infertility?

**DOI:** 10.7759/cureus.30310

**Published:** 2022-10-14

**Authors:** Maria Jose Calero, Maria Resah B Villanueva, Narges Joshaghani, Nicole Villa, Omar Badla, Raman Goit, Samia E Saddik, Sarah N Dawood, Ahmad M Rabih, Aishwarya Raman, Manish Uprety, Ahmad Mohammed, Lubna Mohammed

**Affiliations:** 1 Obstetrics and Gynecology, California Institute of Behavioral Neurosciences and Psychology, Fairfield, USA; 2 Research, California Institute of Behavioral Neurosciences and Psychology, Fairfield, USA; 3 Psychiatry and Behavioral Sciences, California Institute of Behavioral Neurosciences and Psychology, Fairfield, USA; 4 Internal Medicine, California Institute of Behavioral Neurosciences and Psychology, Fairfield, USA; 5 General Surgery, California Institute of Behavioral Neurosciences and Psychology, Fairfield, USA; 6 Pediatrics, California Institute of Behavioral Neurosciences and Psychology, Fairfield, USA

**Keywords:** pregnancy, obstetrical outcomes, pregnancy outcomes, infertility, fertility, adenomyosis

## Abstract

Adenomyosis is a disease related to the presence of endometrial glands and stromal cells within the uterine myometrium that used to be linked to females that are more than 40 years old and multiparous. Nowadays, females are delaying their pregnancies to their third or fourth decade, and as diagnostic approaches evolve, the disease has become a common problem for females who desire pregnancy. The aim of this study is to identify the physio-pathological factors by which adenomyosis causes infertility and pregnancy complications, as well as the possible results from infertility treatments and the most common pregnancy complications that females with adenomyosis face.

A systematic review based on a systematic search from PubMed, Cochrane, and ScienceDirect databases from the past five years was done. Papers with free full text available were subject to the removal of duplicates, screening for relevant titles and abstracts, and a quality assessment to identify the risk of bias (RoB). A total of 10 papers were selected for this study; they include systematic reviews and meta-analyses, cohorts, literature review, and a case-control study.

After the review of the data, we conclude that infertility may be due to several factors that impair adequate sperm mobility through the uterus and an impaired implantation of a product. After some fertility treatments were performed, females with adenomyosis had a lower rate of clinical pregnancy. The pregnancy complications such as preterm delivery and hypertension problems related to pregnancy had an increased risk for females with adenomyosis, while for others such as intrauterine fetal death and gestational diabetes, the information is still controversial. The main limitation of this study was the lack of information of physio-pathological-related information probably due to only including data from the past five years.

## Introduction and background

Adenomyosis is a benign disorder of the uterus described as the presence of endometrial glands and stromal cells within the uterine myometrium. Its clinical presentation is with non-specific symptoms related to the enlargement of the uterus, abnormal uterine bleeding, pelvic pain, menorrhagia, and infertility [1]. Until recently, it was believed that adenomyosis was a condition observed in multiparous females over 40 years old, probably because the diagnosis was made after a hysterectomy with the histopathological study of the uterus [2]. However, newer methods of diagnosis such as magnetic resonance imaging (MRI) and transvaginal ultrasound (TUS) make it possible to have an earlier diagnosis in younger patients and to describe various presentations of the disease. This helps us to find possible etiologies and continue proposing treatments to address symptoms such as infertility. As mentioned by Kobayashi et al. in their very recently published article “Clinicopathological features of different subtypes in adenomyosis: focus on early lesions,” in 2021, “Diffuse adenomyosis may also develop in younger nulligravid women (early 20 years) than previously thought. Thus, adenomyosis is not a disease of the elderly” [1].

As defined by the American College of Obstetricians and Gynecologists (ACOG), “Infertility is the failure to achieve a successful pregnancy after 12 months or more of regular unprotected intercourse, and it affects approximately 12% of the reproductive-aged population” [3]. The cause of infertility in adenomyosis is not well understood; many theories are being studied and described, and many approaches to reach desired pregnancy are being experimented. Females with fertility problems may undergo gonadotropin-releasing hormone (GnRH) analog therapy for the downregulation of the pituitary function, which produces a reduction of adenomyosis since the lesion is sensitive to estrogen, conservative surgery (adenomyomectomy), and assisted reproductive technology (ART), which are all “fertility treatments in which eggs or embryos are handled” [4], or a combined therapy [5].

Nowadays, as new diagnostic methods have been developed, females are postponing their first pregnancy to their late third or fourth decade of life, and as advanced infertility treatments are becoming available, there is an increase of pregnancies complicated by adenomyosis that obstetricians are facing [6]. Thus, there is a recent interest in the possible methods for conceiving and the potential adverse outcomes in these pregnancies, such as miscarriages, preterm deliveries, late pregnancy complications, and small for gestational age (SGA) products [7]. Nevertheless, there is little information about this data as it’s recently been reported; hence in this study, we are looking to understand the physiopathology behind infertility in females with adenomyosis, the results of treatment options available for infertility or subfertility, and the possible pregnancy complications including preterm delivery, pre-eclampsia, gestational diabetes, and intrauterine fetal death, by doing a systematic analysis from the newest available studies in the past five years.

Methods

This systematic review was performed following the Preferred Reporting Items for Systematic Reviews and Meta-Analyses (PRISMA) 2020 [8]. A systematic search was conducted in three databases, PubMed, ScienceDirect, and Cochrane; all papers from the last five years were included from January 2017 to April 2022; the last-day access to the databases was the April 15; gray literature was not included. Studies selected as eligible for the review must be written in the English language, be only human studies, and have free full text available. Inclusion criteria for the references are studies that include females of fertile age from 19 to 64 years old and females with adenomyosis as the study group or studies about the pathophysiology of adenomyosis. Exclusion criteria are females with infertility or subfertility related or caused by any other condition than adenomyosis, such as uterus abnormalities or ovarian dysfunction. The search strategy for Cochrane and ScienceDirect databases used the Boolean scheme. For the PubMed database, a Medical Subject Heading (MeSH) strategy was applied. The search strategy is explained in Table 1.

**Table 1:** Systematic search strategy and results MeSH: Medical Subject Headings; MEDLINE: Medical Literature Analysis and Retrieval System Online

Database	Search strategy	Filter applied	Search results
PubMed (and MEDLINE)	MeSH: Infertility OR (“Infertility, Female/physiology” {Majr} OR “Infertility, Female/statistics and numerical data” {Majr} OR “Infertility, Female/therapy” {Majr}) OR Pregnancy OR (“Pregnancy/abnormalities” {Majr} OR “Pregnancy/complications” {Majr} OR “Pregnancy/statistics and numerical data” {Majr}) AND Adenomyosis OR (“Adenomyosis/pathology” {Majr} OR “Adenomyosis/physiopathology” {Majr})	Year: 2017-2022; text availability: free full text; publication date: five years; species: humans; age: adults 19-44 years and middle age 45-64 years	71
Cochrane	Keywords: Infertility OR Fertility treatment OR Pregnancy OR Pregnancy/Obstetrical outcomes AND Adenomyosis	Publication date: from January 1, 2017, to August 4, 2022	279
ScienceDirect	Keywords: Infertility AND Fertility treatment AND Pregnancy AND Pregnancy/Obstetrical outcomes AND Adenomyosis	Years: 2017, 2018, 2019, 2020, 2021, and 2022	161

All references were grouped in an external reference manager, EndNote (Clarivate, Philadelphia PA), where duplicates were removed and went through screening based on titles and abstracts. Additional eligibility of the references was done by reading the full articles by two authors of the study, Villa and Calero. Finally, all remaining references were subject to a quality assessment from different checklists; a score of at least 70% was required.

Results

**Figure 1:**
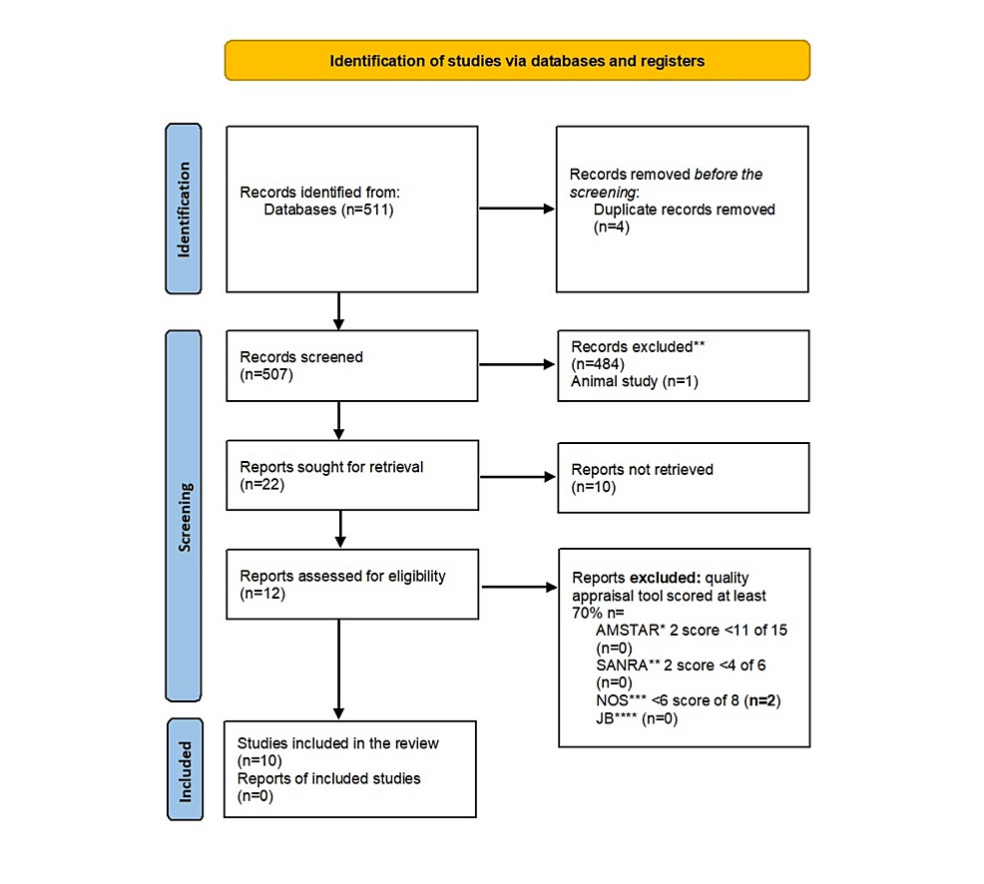
PRISMA chart From Page MJ, McKenzie JE, Bossuyt PM, et al.: The PRISMA 2020 statement: an updated guideline for reporting systematic reviews. BMJ. 2021, 372:n71. 10.1136/bmj.n71 [8] PRISMA: Preferred Reporting Items for Systematic Reviews and Meta-Analyses *Assessment of Multiple Systematic Reviews, **Scale for the Assessment of Narrative Review Articles, ***Newcastle-Ottawa Scale, ****Joanna Briggs reviewers

The systematic search yielded a total of 511 papers, 71 retrieved from PubMed, 279 from Cochrane, and 161 from ScienceDirect. After looking for duplicates, four papers were removed; the remaining 507 papers went through a screening for relevant information from titles and abstracts where 484 papers were not relevant for this study, and one study conducted in animals was removed. For the remaining 22 papers, the full-text version of these papers was read by two authors, Villa and Calero; 10 papers that did not meet the inclusion or exclusion criteria or the full text was not available were removed. Finally, the remaining 12 papers were subject to a quality assessment where if only a score of 70% was reached, the paper was accepted. Figure 1 shows a flow chart of the selection process of the papers.

The quality assessment or risk of bias (RoB) assessment of the final selected papers was done with the following assessments tools, explained on Table 2.

**Table 2:** Risk of bias assessment of papers included in this study AMSTAR: Assessment of Multiple Systematic Reviews; PICO: population, intervention, comparison, outcome; SANRA: Scale for the Assessment of Narrative Review Articles; JBI: Joanna Briggs Institute reviewers

Quality assessment tool	Type of study	Items and characteristics	Minimum accepted score (>70%) over total	Accepted studies
AMSTAR	Systematic review and meta-analysis	Did the research questions and inclusion criteria for the review include the components of PICO? Did the report of the review contain an explicit statement that the review methods were established prior to the conduct of the review, and did the report justify any significant deviations from the protocol? Did the review authors explain their selection of the study designs for inclusion in the review? Did the review authors use a comprehensive literature search strategy? Did the review authors perform study selection in duplicate? Did the review authors perform data extraction in duplicate? Did the review authors provide a list of excluded studies and justify the exclusions? Did the review authors describe the included studies in adequate detail? Did the review authors use a satisfactory technique for assessing the risk of bias (RoB) in individual studies that were included in the review? Did the review authors report on the sources of funding for the studies included in the review? If meta-analysis was justified, did the review authors use appropriate methods for the statistical combination of the results? If meta-analysis was performed, did the review authors assess the potential impact of RoB in individual studies on the results of the meta-analysis or other evidence synthesis? Did the review authors account for RoB in individual studies when interpreting/discussing the results of the review? Did the review authors provide a satisfactory explanation for, and discussion of, any heterogeneity observed in the results of the review? If they performed quantitative synthesis, did the review authors carry out an adequate investigation of publication bias (small study bias) and discuss its likely impact on the results of the review? Did the review authors report any potential sources of conflict of interest, including any funding they received for conducting the review? Scored as yes or no. Partial yes was considered as a point.	12/16	Bruun et al. (2018) [7], Harmsen et al. (2019) [9], Nirgianakis et al. (2021) [10]
SANRA	Narrative reviews	The justification of the article’s importance to the readership, statement of concrete aims or formulation of questions, description of the literature search referencing scientific reason, and appropriate presentation of data, scored as 0, 1, or 2.	5/6	Vannuccini and Petraglia (2019) [2]
Newcastle-Ottawa Scale	Cohort and case-control	The representativeness of the exposed cohort, the selection of the non-exposed cohort, the ascertainment of exposure, demonstration that the outcome of interest was not present at the start of study, and the comparability of cohorts on the basis of the design or analysis. The assessment of outcome was followed up long enough for outcomes to occur. The adequacy of follow-up of cohorts. Scoring was done by placing a point on each category, scored as 0, 1, or 2. *Maximum of two points are allotted in this category.	6/8	Harada et al. (2019) [11], Kim et al. (2019) [12], Shinohara et al. (2020) [6], Yamaguchi et al. (2019) [13], Zhang et al. (2021) [14]
JBI	Case report	Is it clear in the study what is the “cause” and what is the “effect”? Were the participants included in any comparisons similar? Were the participants included in any comparisons receiving similar treatment/care other than the exposure or intervention of interest? Was there a control group? Were there multiple measurements of the outcome both pre- and post-intervention/exposure? Was follow-up complete, and if not, were differences between groups in terms of their follow-up adequately described and analyzed? Were the outcomes of participants included in any comparisons measured in the same way? Were outcomes measured in a reliable way? Was appropriate statistical analysis used?	7/9	Kwack et al. (2021) [15]

Only two studies were removed for not reaching a minimum score of 70%. A Total of 10 papers were accepted into the study; Table 3 summarizes the characteristics of systematic reviews, and Table 4 summarizes the characteristics, cohorts, case-controls, case report, and literature review included.

**Table 3:** Included systematic reviews characteristics MEDLINE: Medical Literature Analysis and Retrieval System Online

Author	Year	Type of study	Databases reviewed	Number of studies included	Total participants	Inclusion (I) and exclusion (E) criteria	Main conclusion
Bruun et al. [7]	2018	Systematic review and meta-analysis	PubMed and Embase	21	2,517, 516 females	I: English language studies, epidemiological studies, observational studies, and studies investigating the associations between endometriosis or adenomyosis and the outcomes of preterm delivery and/or small gestational age (SGA). E: meta-analyses, case reports, and studies with lack of relevance (were not compared with results for females without endometriosis or adenomyosis) or insufficient data reporting and meta-analysis if the associations were not reported as crude or adjusted odds ratio (OR) or relative risks with 95% confidence interval (CI) or if the authors had not provided data that could be used to calculate crude OR with 95% CI.	“Women with adenomyosis is associated with an increased risk of preterm delivery and small-for gestational-age child compared to women without the disease” [7].
Harmsen et al. [9]	2019	Systematic review	PubMed and Embase	First search, 20; second search: nine	Not specified	First search: I: laboratory studies carried out on the endometrium of premenopausal females, original cohort, case-control studies, prospective clinical trials that investigated the presence of angiogenesis in the endometrium of adenomyotic patients, full papers, peer-reviewed journals, focus on markers that indicate the presence of angiogenesis, written in English. E: case reports, studies on cells cultured in vitro not taken from females with adenomyosis or studies that did not compare the endometrium of adenomyosis with the endometrium of control patients without adenomyosis or uterine fibroids.	Increased angiogenesis plays a role in the pathogenesis of adenomyosis and predicts preterm delivery in pregnancies with adenomyosis [9].
Nirgianakis et al. [10]	2020	Systematic review and meta-analysis	MEDLINE, PubMed, and Cochrane	17	Not specified	I: controlled studies where both cases (females with adenomyosis) and controls were assessed, the description of the method of diagnosis of adenomyosis, and the existence of data on fertility and pregnancy or neonatal outcomes. E: data from sources other than original full publications.	“Adenomyosis was significantly associated with a lower clinical pregnancy rate and increased obstetrical complications” [10].

**Table 4:** Included studies characteristics ART: assisted reproductive technology; IVF: in vitro fertilization; GnRH: gonadotropin-releasing hormone

Author	Country and year	Type of study	Number of subjects	Main relevant topic for this study	Main conclusion relevant for this study
Yamaguchi et al. [13]	Japan, 2019	Prospective cohort	314	Fertility treatment and pregnancy outcomes	“Pregnancy with adenomyosis was associated with preterm birth, low birthweight and small for gestational age neonates” [13].
Harada et al. [11]	Japan, 2019	Prospective cohort	96,655	Pregnancy outcomes	“Women with adenomyosis have an increased risk for obstetrical outcomes in pregnancies conceived naturally or after treatment infertility excluding ART therapy” [11].
Kim et al. [12]	Taiwan, 2019	Retrospective cohort	57	Pregnancy complication physiopathology	“Uterine wall thickness measured at second trimester predicts preterm deliveries in pregnancies with adenomyosis [12].
Vannuccini and Petraglia [2]	2019	Literature review	-	Infertility pathophysiology and pregnancy outcomes	Infertility causes in adenomyosis may be caused by a disrupted sperm mobility in the uterus and impaired implantation [2].
Shinohara et al. [6]	Japan, 2020	Case-control	61	Pregnancy outcomes	Patients with adenomyosis have an increased incidence in obstetrical complications [6].
Zhang et al. [14]	China, 2021	Matched case-control	65	Fertility treatment	Adenomyosis has adverse influences in pregnancy outcomes of IVF patients undergoing the long protocols of GnRH [14].
Kwack et al. [15]	Taiwan, 2021	Case reports	466	Infertility treatment and pregnancy outcomes	“Delivery of pregnant women who received adenomyomectomy can obtain safe perinatal outcomes…” [15].

## Review

In this systematic review, we aimed to gather the relevant data available from the past five years about four main parameters that appear as subheadings in this section, and they are infertility physiopathology, the physiopathology of pregnancy complications, fertility treatment, and pregnancy outcomes or complications. For the two subheadings that mention physiopathology, we are looking to explain by which physiological mechanisms adenomyosis causes infertility and pregnancy complications. For the fertility treatment subheading, we collect the results from fertility treatments tested in females with adenomyosis and if available their comparison with results in females without the disease. For the last topic and subheading, pregnancy outcomes or complications, we draw data that compares preterm delivery, pre-eclampsia and pregnancy-induced hypertension, gestational diabetes, and intrauterine fetal death and miscarriage in females with adenomyosis with females without adenomyosis.

Infertility physiopathology

From the 10 papers included in this study, two papers had pertaining information regarding the pathophysiology of infertility. One systematic review concludes that females with adenomyosis have reduced anti-angiogenic and the overexpression of angiogenic markers such as vascular endothelial growth factor leading to an increase in abnormal angiogenesis that impairs the receptivity of the embryo in the uterus, a possible cause for subfertility [9]. Nevertheless, a literature review explained a more detailed information stating that the presence of adenomyosis causes an abnormal contractility of the uterus and therefore an arrhythmic and disrupted peristalsis, affecting sperm transport [2]. When exploring more about this result, we found in a different review that the peristalsis may be enhanced by a pathological transportation of calcium in the myometrium cells causing irregular and dysfunctional hyperactivity of the muscle [16]. Another possible cause for infertility mentioned by the literature review is that as the disorganized tissue of adenomyosis has molecular alterations, it triggers increased inflammatory markers and stress, as well as decreased the expression of adhesion molecules and implantation markers affecting the implantation of a product [2].

As we can see, the etiology of infertility caused by adenomyosis includes multiple components that are being described in the literature as abnormal motility of the uterus and receptivity of the embryo. However, the data extracted from the included studies is limited, possibly due to the small amount of paper dedicated to this topic.

Fertility treatment

Three studies were found on this topic, a systematic review and meta-analysis, a matched case-control study, and a case report. The systematic review and meta-analysis, which separated the studies of ART by their pituitary downregulation protocol with GnRH, found that ultralong or modified ultralong protocols had no significant difference in clinical pregnancy rate nor did the group of long protocol mixed with multiple protocols [10]. However, they did find a significant difference for the short regulation protocol showing a lower clinical pregnancy rate in patients with adenomyosis [10].

In the matched case-control study where the long protocol with GnRH followed by ART was studied, they did find a significant difference of a lower clinical pregnancy rate (47.06% versus 64.42%, P=0.028) [14] and a lower implantation rate (31.91% versus 46.74%, P=0.005) [14]. Compared to females without adenomyosis, also, a higher rate of spontaneous abortion (33.33% versus 13.43%, P=0.034) was found [14].

Nonetheless, data varies depending on the length of GnRH protocols, and we can find that at some point, they conclude that females with adenomyosis may have a lower clinical pregnancy rate than females without adenomyosis. This could be explained by difficulties in implantation or spontaneous abortion caused by the increased levels of estrogen that a pregnant female experiences after being on a pituitary downregulation, which restores the growth of adenomyosis [14].

For the surgical approach of adenomyomectomy, only one case report study was found, where 466 patients were followed after the procedure and only 22 were selected as they got pregnant, 17 required ART with the technique of in vitro fertilization and five of them conceived naturally. It was found that in all the patients that had laparoscopic surgery, four conceived naturally, and the rest had laparotomic surgery [15]. Even though this data comes from a small number of patients, it is helpful in giving us an idea of how the surgical approach works due the limited data available.

Pregnancy complication physiopathology

The pathophysiology of pregnancy complications was also limited due to data found in only one of the studies included, as well as with pathophysiology on infertility. This study is a retrospective cohort that reviewed 57 pregnancies of females with adenomyosis with preterm deliveries and found that in 24.5% of these females, their uterine wall thickness at the second trimester was significantly increased compared to the at-term delivery uterine wall thickness; they suggest that it correlates with increased myometrial stiffness and the production of prostaglandins [12].

Pregnancy outcomes/complications

Preterm Delivery (<37 Weeks)

Preterm delivery or birth is defined by the Centers for Disease Control and Prevention (CDC) as when a baby is born before the 37 weeks of gestation are completed, and “In 2020, preterm birth affected 1 of every 10 infants born in the United States” [17]. Two systematic reviews and meta-analysis that reviewed six and four papers, respectively, on this topic found out that the risk of preterm delivery in patients with adenomyosis is significantly higher and than females without the disease [10,7]. Similarly, two prospective cohorts performed in Japan stated the same conclusion of an increased risk for preterm delivery in females with adenomyosis, the first by multiple logistic regression analysis [13] adjusted for characteristics such as maternal age, smoking status, the method of conception (ART), primiparity, the coexistence of fibroids or endometriosis, and body mass index (BMI) before pregnancy and the second [11] adjusted for females with ART treatments. Lastly, a case-control study also revealed an increased risk [6]. Therefore, it is well stated in many published papers included in this study that the presence adenomyosis in females leads to an increased risk for preterm delivery as a pregnancy complication.

One of the cohorts from Japan also found an increased risk for severe preterm delivery <32 weeks (adjusted odds ratio {aOR}: 3.63; 95% confidence interval (CI): 1.34-9.83) adjusted for ART treatment [11]. However, it was not the case for the systematic review results (OR: 2.20; 95% CI: 0.82-5.89), which did not evidence a higher risk in severe preterm delivery for females with adenomyosis [10]. The data is still controversial on how adenomyosis affects the risk for severe preterm deliveries since we had two papers with information about this topic and each had different results.

Pre-eclampsia and pregnancy-induced hypertension

From the systematic review and meta-analysis by Nirgianakis et al., it also found an increase in pre-eclampsia in females with adenomyosis [10]. In the same way, a large study from Japan adjusted for ART treatment pregnancies [11]. For the risk of pregnancy-induced hypertension, the systematic review previously stated that only one article showed an increase (OR: 3.11; 95% CI: 1.10-8.79) [10]; this one article is also included in our study, so the result will not be stated again, as it comes from the same source [13].

A case-control study found an increased risk for hypertensive disorders of pregnancy as a whole (OR: 2.68; 95% CI: 1.06-6.80) [6]. The information on this topic is limited because the results we are getting from one paper come as a review of another study that is also included in this study; nevertheless, the data is consistent with an increased risk for hypertension disorders during pregnancy in females with adenomyosis.

Gestational diabetes

Again, a systematic review [10] displayed only one paper with data about gestational diabetes mellitus, which comes from a paper included in this study [13]; therefore, it will only be presented once; although the data shows an increased risk of diabetes mellitus, a case-control study did not find a significant increase in the risk for gestational diabetes [10,6].

Intrauterine fetal death and miscarriage

A systematic review and meta-analysis showed that there was not a significant increase in intrauterine fetal death in females with versus without adenomyosis [10]. In contrast, as previously explained infertility treatment results, females undergoing long GnRH protocol and later ART did have a higher rate of miscarriage [14].

As we can see, information in this topic is still controversial due to different results obtained in the papers included in this study. A literature review included in this study showed data from a systematic review and meta-analysis that reported a rate of 31% of miscarriages in females with adenomyosis compared to 12.1% in females without adenomyosis with a “relative risk (RR) 2.12, 95% confidence interval (CI) 1.20-3.75” [2]. In Table 5, we summarize the most relevant data considered for this study, from each included paper.

**Table 5:** Summary of collected data GnRH: gonadotropin-releasing hormone; OR: odds ratio; ART: assisted reproductive technology; BMI: body mass index; aOR: adjusted odds ratio

Topic	Relevant data	Source author and year
Infertility physiopathology	Increased angiogenesis: impairs the receptivity of the embryo in the uterus.	Harmsen et al., 2019 [9]
	Abnormal contractility of the uterus and disrupted peristalsis affect sperm transport. Increased inflammatory markers and decreased expression of adhesion molecules and implantation markers impair the implantation of a product in the uterus.	Vannuccini and Petraglia, 2019 [2]
Fertility treatment	Short pituitary downregulation with GnRH protocol showed a lower clinical pregnancy rate in patients with adenomyosis (OR: 0.34; 95% confidence interval (CI): 0.20-0.57), not the same for long protocol.	Nirgianakis et al., 2021 [10]
	Long GnRH protocol leads to a lower clinical pregnancy rate (47.06% versus 64.42%, P=0.028), lower implantation rate (31.91% versus 46.74%, P=0.005), and a higher rate of spontaneous abortion (33.33% versus 13.43%, P=0.034) in females with adenomyosis compared to females without adenomyosis.	Zhang et al., 2021 [14]
	Surgical procedures from 22 females with adenomyosis, 17 required ART with the technique of in vitro fertilization and five of them conceived naturally. All the patients that had laparoscopic surgery conceived naturally; the rest had laparotomic surgery.	Kwack et al., 2021 [15]
Pregnancy complication physiopathology	Uterine wall thickness in the second trimester of 57 pregnancies was significantly increased compared to the at-term delivery uterine wall thickness (4.49±1.62 cm versus 3.05±1.6 cm, P=0.004), which correlates with increased myometrial stiffness and production of prostaglandins.	Kim et al., 2019 [12]
Pregnancy outcomes/complications	Preterm delivery: the risk of preterm delivery in patients with adenomyosis is significantly higher (OR: 2.65; 95% CI: 2.07-3.39)	Nirgianakis et al., 2021 [10]
	The risk of preterm delivery in patients with adenomyosis is significantly higher (OR: 3.09; 95% CI: 1.88-5.09)	Bruun et al., 2018 [7]
	The risk of preterm delivery in patients with adenomyosis is significantly higher (aOR: 2.49; 95% CI: 1.89-3.41) adjusted for characteristics such as maternal age, smoking status, the method of conception (ART), primiparity, the coexistence of fibroids or endometriosis, and BMI before pregnancy.	Yamaguchi et al., 2019 [13]
	The risk of preterm delivery in patients with adenomyosis is significantly higher (aOR: 2.95; 95% CI: 2.14-4.09) adjusted for females with ART treatments. The risk for severe preterm delivery <32 weeks in patients with adenomyosis is significantly higher (aOR: 3.63; 95% CI: 1.34-9.83) adjusted for ART treatment.	Harada et al., 2019 [11]
	Pre-eclampsia and pregnancy-induced hypertension: increased risk of pre-eclampsia in females with adenomyosis (OR: 4.32; 95% CI: 1.68-11.09).	Nirgianakis et al., 2021 [10]
	The increased risk of pre-eclampsia in females with adenomyosis (aOR: 1.86; 95% CI: 1.11-3.14) [9] adjusted for ART treatment pregnancies.	Harada et al., 2019 [11]
	The increased risk of pregnancy-induced hypertension in females with adenomyosis (OR: 3.11; 95% CI: 1.10-8.79).	Yamaguchi et al., 2019 [13]
	The increased risk of hypertensive disorders of pregnancy in females with adenomyosis (OR: 2.68; 95% CI: 1.06-6.80).	Shinohara et al., 2020 [6]
	Gestational diabetes: the increased risk of gestational diabetes mellitus in females with adenomyosis (OR: 0.11; 95% CI: 0.02–0.85).	Nirgianakis et al., 2021 [10]
	There was no increase in gestational diabetes in females with adenomyosis (OR: 1.83; 95% CI: 0.80-4.25).	Shinohara et al., 2020 [6]
	Intrauterine fetal death and miscarriage: not a significant increase in intrauterine fetal death in females with adenomyosis (OR: 1.43; 95% CI: 0.34-6.04).	Nirgianakis et al., 2021 [10]
	Thirty-one percent of miscarriages in females with adenomyosis compared to 12.1% in females without adenomyosis with a “relative risk (RR) 2.12, 95% confidence interval (CI) 1.20-3.75.”	Vannuccini and Petraglia, 2019 [2]

Limitations

The biggest limitation faced in this study was that, including only five years of publication, we may be losing some important information described in the past years when research regarding adenomyosis was focused on some other categories such as the pathophysiology of infertility or the pathophysiology of pregnancy complication, leading to limited information in some topics of this study. Nevertheless, in the past five years, there is much recent and updated information about adenomyosis, highlighting the importance of this systematic review.

## Conclusions

To conclude, we can state that even though there is limited information about the pathophysiology of infertility and the pathophysiology of pregnancy outcomes, infertility is likely related to impaired mobility of sperms through the uterus and a difficult implantation of a product in the uterus of females with adenomyosis. While for pregnant patients, their outcomes may be influenced by the thickness of their uterine walls. The fertility treatment for females with adenomyosis is broad, but there is more information about ART after pituitary downregulation with GnRH, where the rate of clinical pregnancy achieved is still controversial since studies find both a lower rate and no difference between females with adenomyosis and females without it. Finally, data supporting an increased risk of preterm delivery, an increased risk for hypertension problems related to pregnancy, and a lower rate of pregnancy in females with adenomyosis is consistent. In other results, such as intrauterine fetal death and gestational diabetes, the information is still controversial, with some studies pointing in different directions. As the incidence of fertile females with adenomyosis continues to increase, studying the parameters included in this systematic review is important, since it can help the medical community provide accurate details about the disease and treatment options for these patients.
